# Predicting drug side-effect profiles: a chemical fragment-based approach

**DOI:** 10.1186/1471-2105-12-169

**Published:** 2011-05-18

**Authors:** Edouard Pauwels, Véronique Stoven, Yoshihiro Yamanishi

**Affiliations:** 1 Mines ParisTech, Centre for Computational Biology, 35 rue Saint-Honoré, F-77305 Fontainebleau Cedex, France; 2 Institut Curie, F-75248, Paris, France; 3 INSERM U900, F-75248, Paris, France

## Abstract

**Background:**

Drug side-effects, or adverse drug reactions, have become a major public health concern. It is one of the main causes of failure in the process of drug development, and of drug withdrawal once they have reached the market. Therefore, *in silico *prediction of potential side-effects early in the drug discovery process, before reaching the clinical stages, is of great interest to improve this long and expensive process and to provide new efficient and safe therapies for patients.

**Results:**

In the present work, we propose a new method to predict potential side-effects of drug candidate molecules based on their chemical structures, applicable on large molecular databanks. A unique feature of the proposed method is its ability to extract correlated sets of chemical substructures (or chemical fragments) and side-effects. This is made possible using sparse canonical correlation analysis (SCCA). In the results, we show the usefulness of the proposed method by predicting 1385 side-effects in the SIDER database from the chemical structures of 888 approved drugs. These predictions are performed with simultaneous extraction of correlated ensembles formed by a set of chemical substructures shared by drugs that are likely to have a set of side-effects. We also conduct a comprehensive side-effect prediction for many uncharacterized drug molecules stored in DrugBank, and were able to confirm interesting predictions using independent source of information.

**Conclusions:**

The proposed method is expected to be useful in various stages of the drug development process.

## Background

Drug side-effects, or adverse drug reactions, have become a major public health concern. It is one of the main causes of failure in the process of drug development, and of drug withdrawal once they have reached the market. As an illustration of the extent of this problem, serious drug side-effects are estimated to be the fourth largest cause of death in the United States, resulting in 100,000 deaths per year [1]. In order to reduce these risks, many efforts have been devoted to relate severe side-effects to some specific genetic biomarkers. This so-called pharmacogenomics strategy is a rapidly developing field, especially in oncology [2]. The aim is to prescribe a drug to patients who will benefit from it, while avoiding life threatening side-effects [3].

From the viewpoint of systems biology, drugs can be regarded as molecules that induce perturbations to biological systems consisting of various molecular interactions such as protein-protein interactions, metabolic pathways and signal transduction pathways, leading to the observed side-effects [4]. Actually, the body's response to a drug reflects not only the expected favorable effects due to the interaction with its target, but also integrates the overall impact of off-target interactions. Indeed, even if a drug has a strong affinity for its target, it also often binds to other protein pockets with varying affinities, leading to potential side-effects. This concept has been illustrated by comparing pathways affected by toxic compounds and those affected by non-toxic compounds, establishing links between drug side-effects and biological pathways [5].

Although preclinical *in vitro *safety profiling can be used to predict side-effects by testing compounds with biochemical and cellular assays, experimental detection of drug side-effects remains very challenging in terms of cost and efficiency [6]. Therefore, *in silico *prediction of potential side-effects early in the drug discovery process, before reaching the clinical stages, is of great interest to improve this long and expensive process and to provide new efficient and safe therapies for patients. Expert systems based on the knowledge of human experts have been developed to predict the toxicity of molecules based on the presence or absence of toxic moieties in their chemical structure. For example, they predict potential toxicity such as mutagenicity, but they do not provide prediction for numerous potential side-effects in human [7]. Recently, several computational methods for predicting side-effects have been proposed, and the methods can be categorized into pathway-based approaches and chemical structure-based approaches, which are respectively reviewed below.

The principle of pathway-based approaches is to relate drug side-effects to perturbed biological pathways or sub-pathways because these pathways involve proteins targeted by the drug. In a pioneer work to illustrate this concept, it has been shown that drugs with similar side-effects tend to share similar profiles of protein targets [8]. The authors further exploited this characteristic to predict missing drug targets for known drugs using side-effect similarity. Fukuzaki et al has proposed a method for relating side-effects to cooperative pathways defined as sub-pathways sharing correlated modifications of gene expression profiles in presence of the drug of interest [9]. However, this method requires gene expression data observed under chemical perturbation of the drug. Xie et al developed a method to identify off-targets for a drug by docking this drug into proteins binding pocket similar to that of its primary target has been proposed [10]. The drug-protein interactions with the best docking scores are incorporated to known biological pathways, which allows us to identify potential off-target binding networks for this drug. However, the performance of this method depends heavily on the availability of protein 3D structures and known biological pathways, which limits its large-scale applicability.

The principle of chemical structure-based approaches is to relate drug side-effects to their chemical structures. Scheiber et al developed a method that identifies chemical substructures associated to side-effects [11]. However, this method does not provide an integrated framework to predict side-effects for any drug molecule. Yamanishi et al proposed a method to predict pharmacological and side-effect information using chemical structures, which is then used to infer drug-target interactions [12]. However, the method cannot be applied to predict high-dimensional side-effect profiles.

In the present work, we develop a new method to predict potential side-effect profiles of drug candidate molecules based on their chemical structures, which is applicable on large molecular databanks. A unique feature of the proposed method is its ability to extract correlated sets of chemical substructures (or chemical fragments) and side-effects. This is made possible using sparse canonical correlation analysis (SCCA). To our knowledge, no other computational method has been reported for both predicting drug side-effects and associating these side-effects with the presence of identified chemical substructures. In the results section, we show the usefulness of the proposed method on the prediction of 1385 side-effects in the SIDER database from the chemical structures of 888 approved drugs. These predictions are performed with simultaneous extraction of correlated ensembles formed by a set of chemical substructures shared by drugs that are likely to have a set of side-effects. We also conduct a comprehensive side-effect prediction for many uncharacterized drug molecules stored in DrugBank, and were able to confirm interesting predictions using independent source of information.

## Results

### Data representation

**Figure 1:**
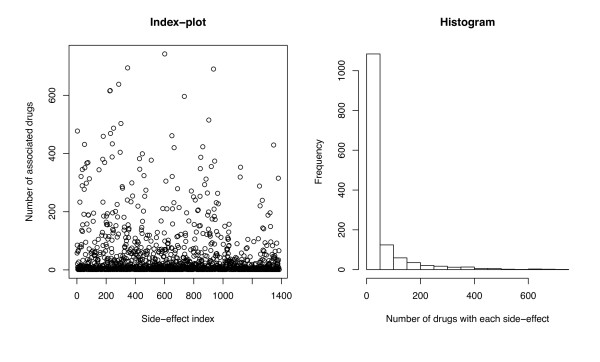
**Characteristics of side-effect data**. The left panel shows the index-plot of the number of associated drugs for each side-effect, and the right panel shows the histogram of the number of associated drugs for each side-effect.

Side-effect keywords were obtained from the SIDER database which contains information about marketed medicines and their recorded adverse drug reactions [13]. This led to build a dataset containing 888 drugs and 1385 side-effect keywords. Each drug was represented by a 1385 dimensional binary profile **y **whose elements encode for the presence or absence of each of the side-effect keywords by 1 or 0, respectively. The left panel in Figure 1 shows the index-plot of the number of associated drugs for each side-effect, and the right panel in Figure 1 shows the histogram of the number of associated drugs for each side-effect. There are 61,102 associations between drugs and side-effect terms in the dataset, and each drug has 68.8 side-effects on average. This dataset is used to evaluate the performance of the proposed methods in this study.

To encode the drug chemical structure, we used a fingerprint corresponding to the 881 chemical substructures defined in the PubChem database [14]. Each drug was represented by an 881 dimensional binary profile **x **whose elements encode for the presence or absence of each PubChem substructure by 1 or 0, respectively. A description of the 881 chemical substructures can be found at the website of PubChem [14]. There are 107,292 associations between drugs and chemical substructures in the dataset, and each drug has 120.8 substructures on average.

The other drug information (e.g., ATC code, drug category, protein target) was obtained from DrugBank [15]. This information is used to ease biological interpretation in the side-effect prediction for uncharacterized drugs.

### Performance evaluation

We applied nearest neighbor (NN), support vector machine (SVM), ordinary canonical correlation analysis (OCCA), and sparse canonical correlation analysis (SCCA) to predict drug side-effect profiles. We also applied random assignment procedure (Random) as a baseline method. For the details of the algorithm of each method, see the Methods section. First we tested five methods: Random, NN, SVM, OCCA and SCCA for their abilities to predict known side-effects profiles by the following 5-fold cross-validation. Drugs in the side-effect data were split into 5 subsets of roughly equal size, each subset was then taken in turn as a test set, and we performed the training on the remaining 4 sets. For accurate comparison, we kept the same experimental conditions, where the same training drugs and test drugs are used across the different methods in each cross-validation fold. We evaluated the performance of each method by the ROC (receiver operating characteristic) curve [16], which is a graphical plot of the sensitivity, or true positive rate, against false positive rate (1-specificity or 1-true negative rate). The ROC curve can be represented by plotting the fraction of true positives out of the positives (true positive rate) vs. the fraction of false positives out of the negatives (false positive rate), where true positives are correctly predicted side-effects and false positives are incorrectly predicted side-effects based on the prediction score for various threshold values above which the output is predicted as positive and negative otherwise.

**Figure 2:**
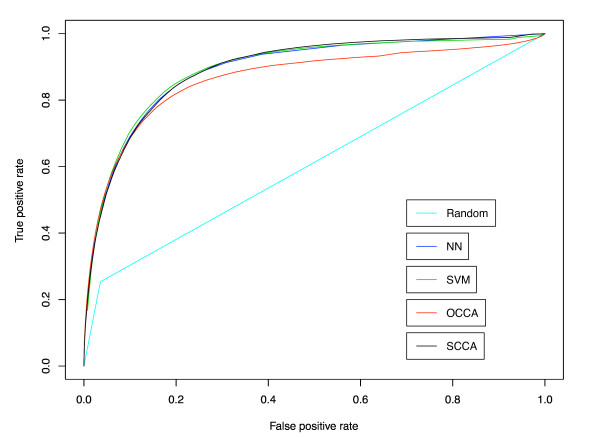
**ROC curves in the 5-fold cross-validation**. Comparison of the performance between nearest neighbor (NN), support vector machine (SVM), ordinary canonical correlation analysis (OCCA) and sparse canonical correlation analysis (SCCA).

**Figure 3:**
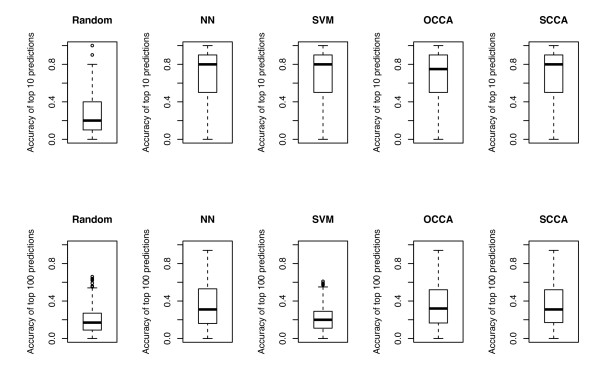
**Boxplot of the prediction accuracy of predicted side-effects for each drug**. Prediction accuract of top 10 ranked predictions (top panels) and top 100 ranked predictions (bottom panels). Comparison of the performance between nearest neighbor (NN), support vector machine (SVM), ordinary canonical correlation analysis (OCCA) and sparse canonical correlation analysis (SCCA).

Figure 2 shows the ROC curves for the five different methods based on the cross-validation experiment, where the prediction scores for all side-effects were merged and a global ROC curve was drawn for each method. Parameters in each method were chosen by using the AUC (area under the ROC curve) score as an objective function. The best result for NN was obtained by the number of neighbors *k *= 50. The best result for SVM was obtained by Gaussian RBF kernel with width parameter *σ *= 0.2 and regularization parameter *C *= 1. The best result for OCCA was obtained by *m *= 20. The best result for SCCA was obtained by the following parameters: *c*_1 _= *c*_2 _= 0.05 and *m *= 20. The resulting AUC scores for Random, NN, SVM, OCCA and SCCA are 0.6088, 0.8917, 0.8930, 0.8651 and 0.8932, respectively. It seems that the proposed SCCA method outperforms OCCA and its performance is at a competitive level with NN and SVM. This result demonstrates the high-performance prediction power of the proposed method on side-effect prediction in practical applications. Next we evaluated the prediction accuracy of predicted side-effects for each drug, which is the ratio of correctly predicted side-effects against the number of predicted side-effects with high prediction scores. Figure 3 shows the boxplots for the prediction accuracies of top 10 ranked predictions (top panels) and top 100 ranked predictions (bottom panels). In the case of top 10 predictions SVM seems to work as well as NN and SCCA, while in the case of top 100 predictions SVM seems to work worse than other methods. This result suggests that SVM-based prediction is useful only for highly ranked predictions.

**Figure 4:**
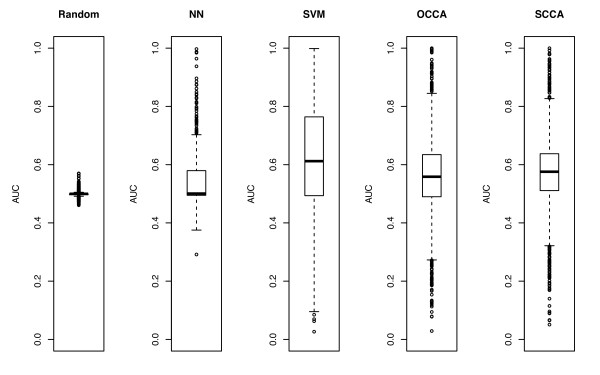
**Boxplot of the AUC (under the ROC curve) scores for individual side-effects**. Comparison of the performance between nearest neighbor (NN), support vector machine (SVM), ordinary canonical correlation analysis (OCCA) and sparse canonical correlation analysis (SCCA).

We also examined the prediction accuracy for individual side-effects. We draw the ROC curve for each side-effect, and computed the AUC score for each side-effect. Figure 4 shows the boxplot representing the distribution of the resulting AUC scores for 1385 side-effects in each method. Parameters in each method were chosen by using the mean of AUC scores as an objective function. The best result for NN was obtained by the number of neighbors *k *= 10. The best result for SVM was obtained by Gaussian RBF kernel with width parameter *σ *= 0.1 and regularization parameter *C *= 1. The best result for OCCA was obtained by *m *= 150. The best result for SCCA was obtained by the following parameters: *c*_1 _= *c*_2 _= 0.2 and *m *= 500. In terms of the mean of the AUC scores, SVM seems to work the best, followed by SCCA, OCCA, and NN, but the AUC scores of SVM is more diverse than those of other methods. Compared with other methods, the difference between good accuracy and bad accuracy is extremely large, which suggests that the prediction success of SVM is not robust and depends on a given side-effect term.

**Figure 5:**
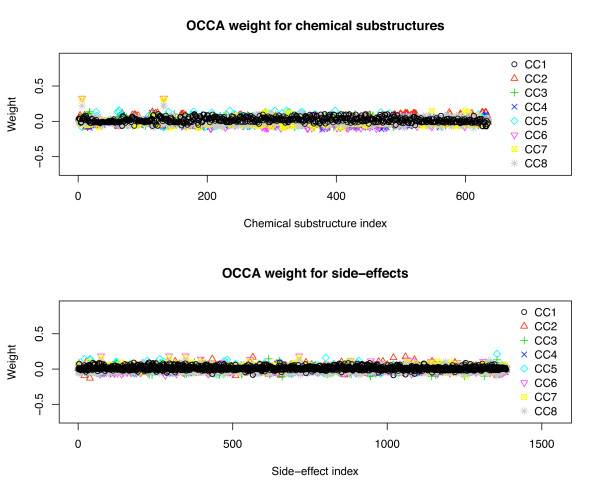
**Index-plot of weight vectors for drug substructures and side-effects in OCCA**. Index-plot of weight vectors for drug substructures (left) and side-effects (right) extracted by ordinary canonical correlation analysis (OCCA).

**Figure 6:**
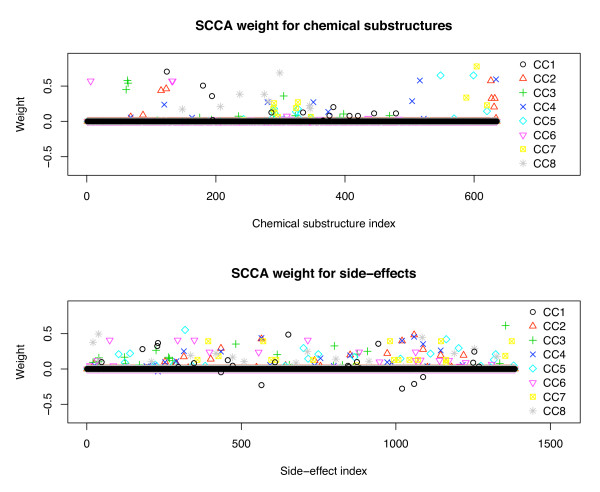
**Index-plot of weight vectors for drug substructures and side-effects in SCCA**. Index-plot of weight vectors for drug substructures (left) and side-effects (right) extracted by sparse canonical correlation analysis (SCCA).

We are also interested in biological interpretability of the outputs of the proposed method to understand the relationship between chemical substructures and side-effects. We focused on OCCA and SCCA, because they are the only methods which can correlate two heterogeneous high-dimentional data sets. We examined the weight vectors for drug chemical substructures and drug side-effects in OCCA and SCCA. Figure 5 shows the index-plot of weight vectors in OCCA, and Figure 6 shows the index-plot of weight vectors in SCCA, where the first eight canonical components are shown. It seems that almost all elements in the weight vectors in OCCA are non-zero and highly variable, while most of the elements in the weight vectors in SCCA are zero in each component, implying that SCCA can select a small number of features as informative drug substructures and side-effects. In practice, we found that it is very difficult to interpret the results when there are too many non-zero weight elements like with OCCA. This result suggests that the proposed SCCA method provides us with more selective and informative correlation between drug substructures and side-effects without loosing performance. This highlights the significant performance of the proposed method in terms of easier interpretation. In addition, it should be pointed out that the other methods NN and SVM do not provide any clue for biological interpretation.

**Figure 7:**
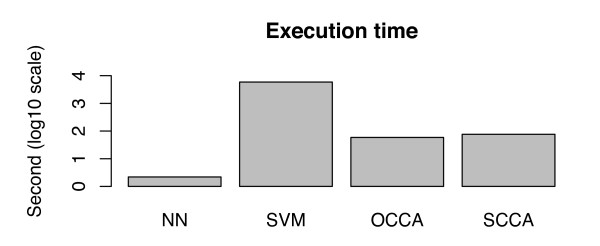
**Computational cost**. Total execution time of the cross-validation experiment for the four methods (log10 scale).

Finally, we investigated the computational cost for each method. All methods were implemented using R software on a Linux with 2.16 GHz Intel Core 2 Duo processor and 8 GB RAM. The total execution times of the cross-validation experiment for NN, SVM, OCCA, and SCCA are 2, 5885, 58, and 76 seconds, respectively. Figure 7 shows the total execution times of the cross-validation experiment between the four different methods in the scale of base10 logarithm. It seems that NN is the fastest, followed by OCCA, SCCA, and SVM. As expected, SVM is extremely slower than the other methods, because it requires individual classifiers for all side-effect keywords (1385 SVM classifiers are required in this study).

### Extracted sets of drug substructures and side-effects

From biological viewpoints, we examined the extracted sets of drug substructures and drug side-effects in each canonical component extracted using SCCA. Note that the other methods (NN, SVM, and OCCA) do not enable us to interpret the biological features. Each component consists of only a small number of substructures and a small number of side-effects that are correlated with each other according to SCCA. For each component, two lists of drugs are provided: one containing drugs with a high score for the associated substructures, and one containing drugs with a high score for the associated side-effects. We examined the results when we used the best parameters which provided the highest AUC for all side-effect terms. Because of space limitation, the results for a few canonical components will be discussed in this paper. The results for all canonical components can be obtained from Additional file 1 in the Supplemental materials or from the web supplement.

A canonical correlation coefficient is computed to evaluate the importance of each component. The p-values for the canonical correlation coefficients of top 20 components considered in the paper are almost zeros. The components with high canonical correlation tend to contain rare substructures present only in very few drugs, which are associated to rare side-effects mainly observed for these drugs. These components contain quite specific substructure/side-effect canonical correlations whose interpretation is straightforward. For example, component 6 associates the presence of a boron atom, only found in the bortezomid molecule in the SIDER database, to a short list of neurological side-effects observed only for this drug. Similarly, component 20 essentially clusters a substructure defined by a carbon atom bearing both a bromide atom and a nitrogen atom. This substructure is found only in the bromocriptine molecule of the SIDER database, with two side-effects observed only for this drug (namely, pregnancy induced hypertension and toxemia of pregnancy).

**Figure 8:**
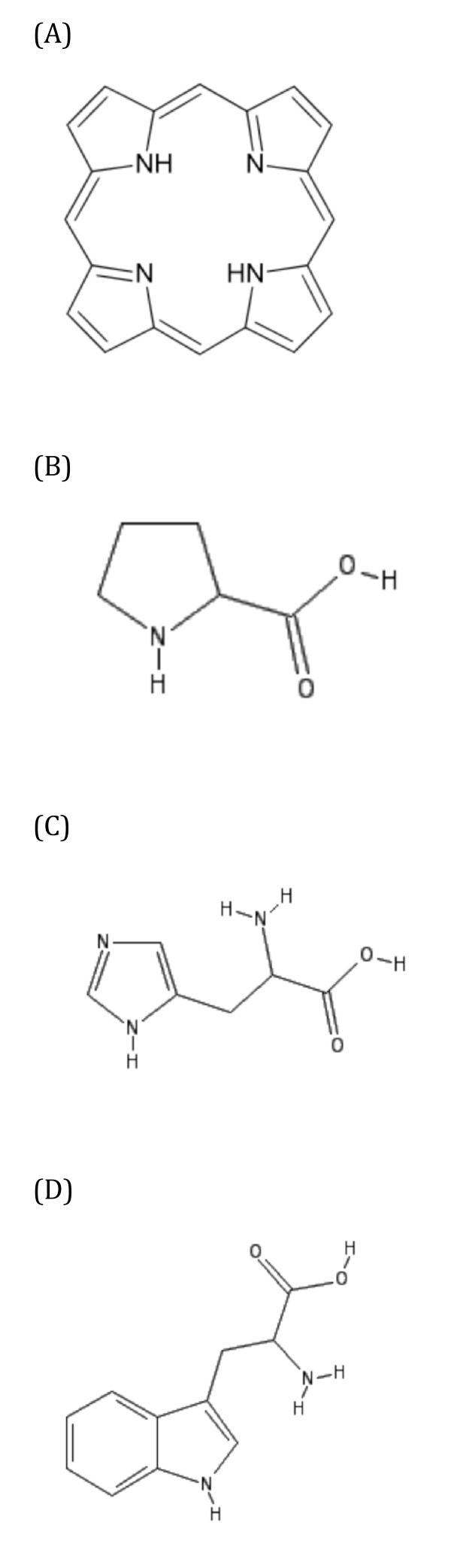
**Nitrogen-containing rings of size 5**. (A) Porphyrin group, (B) Proline residue, (C) Histidine residue, (D) Tryptophane residue.

In the general case of components containing more frequent substructures, drugs that contain these substructures tend to present side-effects associated to this component, but this correspondence is not strict. Reciprocally, most drugs that have high scores for the side-effects contain the chemical substructures of this component, but not all. Analysis of component 18 can illustrate these points. Component 18 has a high canonical correlation of 0.739 (the p-value is almost zero). It contains two substructures, the major one being the presence of "four or more saturated or aromatic nitrogen-containing rings of size 5", associated to four side-effects. This substructure is present in five drugs of the SIDER database: verteporfin, porfimer, goserelin, buserelin, and leuprolide. Verteporfin and porfimer contain a porphyrin group displaying four nitrogen-containing rings of size 5, as shown in Figure 8(A). Goserelin, buserelin, and leuprolide are synthetic 9-residue peptide analogues of the gonadotropin releasing hormone. Their sequences contain amino-acids whose chemical structures present nitrogen-containing rings of size 5, found in side chains of proline, histidine or tryptophane residues, as shown in Figure 8(B), (C) and Figure 8(D).

Overall, four or more nitrogen-containing rings of size 5 are indeed present in their structures. Note however that these rings are different from those of the porphyrin group. Although goserelin, buserelin and leuprolide on the one hand, and verteporfin and porfimer on the other hand, belong to totally unrelated families of molecules, they share common substructures, at least according to their definition in the present study. All drugs from these two families, but verteporfirin, have high scores for side-effects of this component. This result indicates that side-effects of a drug is usually associated to the presence of given substructures, although it may be modulated by the overall molecular structure, as in the case of verteporfirin. This property is also well known in the context of drug structure-activity relationship, which usually depends on given molecular scaffolds, but which is modulated by the presence of additional chemical groups.

**Figure 9:**
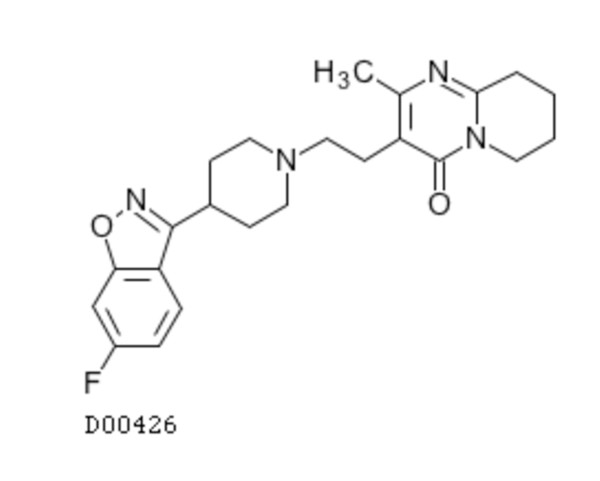
**Chemical structure of risperidone**. Two dimensional graph structure of risperidone.

Reciprocally, all drugs that have high scores for side-effects of component 18 contain the chemical substructures of this component, but risperidone, as shown in Figure 9. Its structure is very different from those of porphyrins or gonadotropin analogues. It is an antagonist of the dopamine and of the serotonine receptors. It belongs to the class of antipsychotic agents (see DrugBank), and its high score for side-effects of component 18 cannot be explained in a straightforward manner.

However, in some cases, we were able to relate such unexpected results to the targets of these drugs, as illustrated by component 13. This component has a canonical correlation of 0.716 (the p-value is almost zero), and contains substructures that are essentially present in proton pump inhibitors used as anti-ulcer agents like omeprazole. It is also present in a small number of drugs from other families like pramipexole (an antiparkinson agent) or riluzone (a neuroprotective agent). As expected, these anti-ulcer agents are found in the high scoring drugs for side-effects in component 13, together with pramipexole and riluzone, although with lower scores. As for component 18, other drugs that do not contain the high scoring substructures of component 13 are however found among high scoring drugs for side-effects in this component. This is the case of ropinirole. Interestingly, ropinirole is an antiparkinson agent that targets the same protein as pramipexole, namely dopamine receptor.

This result suggests that drugs sharing some protein targets may also share some side-effects. It is also consistent with the idea that the global biological effect of a molecule (both beneficial effects and adverse side-effects) is related to its overall profile of protein targets. Taken together, our results indicate that the side-effects of a drug are modulated both by its substructures and by its targets. Note that these two factors are connected since similar molecules tend to share similar protein targets, but this property was not exploited in the present study.

### Comprehensive side-effect prediction for uncharacterized drugs

We then evaluated the interest of the proposed method for prediction of side-effects for uncharacterized drugs. We predicted potential side-effects for drugs in DrugBank for which side-effect information was not available in the SIDER database. We focused on 2883 drugs which are labeled as "small molecules" in DrugBank. We first make general comments on the results and then present more details for a few well-known specific examples. All the prediction results can be obtained from Additional file 2 in the Supplemental materials or from the web supplement.

Very frequent side-effects, such as "headache" or "nausea" are found in SIDER, and they occur with many drugs. These side-effects are not specific, and they do not appear for a well defined drug category. They are the most frequently predicted side-effects, but they hardly appear with the highest prediction scores for a given drug, which is consistent with the fact that they are common reactions. However, we also find more specific side-effects which are related to special types of drugs. For example, steroids may lead to "striae", or "linear atrophy", which results in local dermal structure atrophy and skin depigmentation [17]. Indeed, this keyword is mainly found for steroid molecules in SIDER. The top 30 drugs predicted to have this side-effect are also steroids, which is consistent with literature and training data. Moreover, "global amnesia", a very specific keyword in SIDER, is one of the most striking syndromes in clinical neurology whose underlying causes are not well known [18]. 14 drugs catch a high prediction score for this keyword. Among them, one is anticholesteremic, three are antipsychotics, and the others are experimental molecules whose categories are not known. Therefore, three out of four drugs with known indications are related to cognitive functions, which is consistent with the predicted side-effect nature. Although the accuracy of all the predictions was not discussed here, the results are consistent with the available biological and medical information.

**Figure 10:**
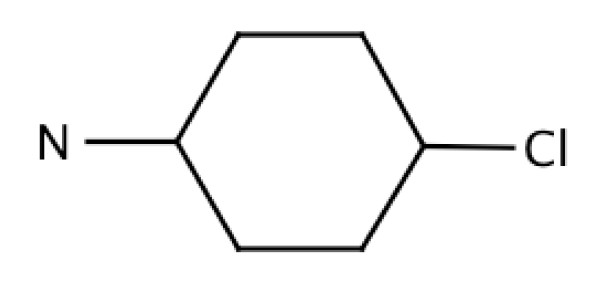
**Rimonabant substructure selected by the proposed method to be a clue of psychoacticity**. The substructure of Rimonabant is selected to be a clue of psychoacticity.

We also checked famous examples of withdrawn drugs. Rimonabant (DB06155 in DrugBank) is an anti-obesity agent. It was rejected for approval in the United States, but it was accepted in Europe in 2006. In october 2008, the European Medicines Agency recommended suspension of its marketing authorization because of serious psychic side-effects, mainly severe depression. Indeed, this drug is active in the central nervous system, which may trigger very broad and complex psychic mechanisms. Consistent with this, in our prediction profile, the "borderline personality disorder" and "posttraumatic stress disorder" keywords are found in the ten top ranking keywords for this drug. In other words, our method would have foreseen potential psychoactivity for rimonabant. Furthermore, the method provides a potential rationale for appearance of these psychotic effets. Rimonabant contains the substructure shown in Figure 10. This substructure is also found in the alprazolam molecule used in the treatment of psychic disorders (a molecule in SIDER). Interestingly, among the 165 molecules of PubChem that also share this substructure and for which pharmacological annotation is available, 40 are classified as "anti-anxiety agents". A reasonable hypothesis to explain rimonabant's severe side-effects may be the presence of this substructure, together with the nature of its protein target (namely, the cannabinoid receptor). Terfenadine (DB00342 in DrugBank) is an anti-allergic agent which was withdrawn by the U.S. Food and Drug Administration in 1997 because of toxic effects on heart rhythm. The "Aortic stenosis" and "aortic valve incompetence" keywords rank 9-th and 11-th among the predicted side-effects for this drug. These related side-effects are known to often lead to arrhythmias [19], as observed for this drug. In this case again, our method would have foreseen potential severe cardiac side-effects.

## Discussion

In this paper we showed the usefulness of the proposed SCCA method in the analysis of chemical structures and side-effects, but there are several limitations on SCCA. One main difficulty of using SCCA is to choose appropriate sparsity parameters and appropriate number of components. High sparsity promoting parameters would lead to an over-sparse model in all the cases, which might be misleading in the interpretation if the degree of sparsity was not tuned carefully. The optimal parameters value depends highly on the definition of the objective function to be investigated in the cross validation. We evaluated both global prediction accuracy, involving all possible drug-sideeffect associations, and local accuracy considering each sideeffect keyword independantly. Those two evaluation procedures did not lead to the same optimal parameter values (it varies between 0 and 1). The definition of an appropriate objective function in the cross-validation is an important issue. There remains much room to develop a more appropriate way to choose the parameters, depending on the goal of the analysis. When the goal is the global accuracy (accuracy for all side-effects for each drug), the sparsity parameter producing the best results tends to be very low, which produces canonical components associated with very few substructures and side-effects as shown in the results section. On the other hand, the goal is the local accuracy (accuracy for individual side-effects), the sparsity parameter producing the best results tends to be relatively high, which produces canonical components associated with a larger number of substructures and side-effects. The extracted features based on SCCA is also influenced by the procedure of data normalization. In this study we normalized two data sets by centering and scaling with unit variance. In our experience, when the scaling is performed on data sets, SCCA tends to extract less frequent features (both side-effects and chemical substructures). On the other hand, when the scaling is not performed on data sets, SCCA tends to extract more frequent features (both side-effects and chemical substructures). Therefore, an appropriate data normalization procedure is supposed to be performed taking into account the objective in practical applications. For example, if the user wants to extract rare features, the scaling is encouraged, but otherwise, the scaling is not necessary.

Another possible statistical method with high interpretability would be a decision tree learner or a rule learner. However, these methods can be applied to only one response variable (one side-effect term in this study). For example, if the decision tree method [20] is applied to the problem addressed in this paper, it requires learning for all side-effects separately. We then need to interpret 1385 resulting trees, so it is quite difficult to make a global interpretation. Note that we have two heterogeneous high-dimensional data sets: drug chemical substructures and drug side-effects, and we are interested in joint extraction of a subset of chemical substructures and a subset of side-effects which are suspected to be correlated with each other. It would be interesting to extend the decision tree framework to analyze the correlation between two heterogeneous high-dimensional data, but it is out of scope in this paper.

The proposed methods depend highly on the pre-definition of chemical substructures, and the terminology of side-effect keywords. Future development could evaluate the performance of using other fingerprints. For example, commercial softwares such as Daylight or Dragon provide drug structure descriptors, and commercial databases such as PharmaPendium provide other side-effect terms. Another interesting research direction is to extract informative chemical sub-structures directly from the raw structured data (e.g., 2D or 3D graph structures for drugs) without using pre-defined feature representation. Recently, a data mining technique has been proposed in order to extract complex graph features, which do not require the pre-definition of feature vectors representing each molecule [21-27]. A promising future work would be an extension of such graph mining techniques in the context side-effect prediction.

## Conclusion

In this paper we proposed a novel method to predict potential side-effect profiles of drug candidate molecules based on their chemical structures using sparse canonical correlation analysis (SCCA). The method is computationally efficient and is applicable on large datasets. The originality of the proposed method lies in the integration of chemical space and pharmacological space in a unified framework, in the extraction of correlated sets of chemical substructures and side-effects, and in the prediction of a large number of potential side-effects at a time. To our knowledge, no previous work gathers all these features.

The proposed method is expected to be useful in various ways and at various stages of the drug development process. At early stages, among several active drug candidates, the method could help to choose the molecules that should further continue the process and those that should be dropped. It could also help to find new indications for known drugs, a process named drug repurposing. Indeed, side-effects of drugs used in a given pathology can be viewed as a beneficial effect in another pathology. Sildenafil is a famous example of such drug repositioning. The method could help to identify chemical substructures of known drugs that might participate in the appearance of a given side-effect. These substructures could be used as building blocks in fragment-based drug discovery approaches [28] for pathologies in which this side-effect could be positively exploited.

## Methods

We propose five possible methods to predict drug side-effect profiles from the chemical structures.

### Random assignment (Random)

To evaluate how difficult the problem considered in this paper is, we apply a random assignment procedure, that is, we use the 0/1 ratio to assign a binary label to each test drug randomly. For example, if the ratio in given training data is 90%, we can assign zero for 90% of examples in test; otherwise 1. This method is used as a baseline method in this study.

### Nearest neighbor (NN)

The most straightforward approach is to apply the nearest neighbor (NN), which predicts a given drug **x **to have the same side-effects as those of the drug (in a training set) whose chemical substructure profile is the most similar. For each query drug, we look for **k **nearest neighbors, and if *k' *of *k *have a side-effect, we assign the prediction score of *k'*/*k *to the query drug. We repeat this procedure for *q *side-effects.

### Support vector machine (SVM)

A more sophisticated approach would be to apply a supervised binary classification method for predicting whether a given drug **x **has a side-effect or not, and repeat this process for all *q *side-effects. The support vector machine (SVM) is a well-known binary classifier, and it has become a popular classification method in bioinformatics [29] and chemoinformatics [21] because of its high-performance prediction ability [30]. We test several kernel functions such as linear kernel, Gaussian RBF kernel with various width parameters, and polynomial kernel with various degree parameters. Note that this strategy needs to construct *q *individual SVM classifiers for *q *side-effects, so it will require considerable computational burden, because *q *is quite huge in practical applications (*q *is 1385 in this study).

### Ordinary canonical correlation analysis (OCCA)

Suppose that we have a set of *n *drugs with *p *substructure features and q side-effect features. Each drug is represented by a chemical substructure feature vector **x **= (*x*_1 _, ⋯, *x_p _*)*^T ^*, and by a side-effect feature vector **y **= (*y*_1 _, ⋯, *y_q _*)*^T ^*.

Consider two linear combinations for chemical substructures and side-effects as *u_i _*= ***α****^T ^***x***_i _*and *v_i _*= ***β****^T ^***y***_i _*(*i *= 1, 2, ⋯, *n*), where ***α ***= (*α*_1_, ⋯, *α_p_*)*^T ^*and ***β ***= (*β*_1 _, ⋯, *β_q _*)*^T ^*are weight vectors. The goal of ordinary CCA is to find weight vectors ***α ***and ***β ***which maximize the following canonical correlation coefficient:(1)

Where  is assumed and *u *(resp. *v*) is called *canonical component *for **x **(resp. **y**) [31].

Let × denote the *n *× *p *matrix defined as *X *= [**x**_1 _, ⋯, **x***_n_*]*^T ^*, and let Y denote the *n *× *q *matrix defined as *Y *= [**y**_1 _, ⋯, **y***_n _*]*^T ^*. The columns of X and Y are assumed to be centered and scaled. Then the maximization problem can be written as follows:(2)

In other high-dimensional problems, it is known that good results can be obtained by treating the covariance matrix as a diagonal matrix [32,33], as suggested in [34]. Therefore, we substitute identity matrices for *X^T ^X *and *Y^T ^Y *, and consider the following optimization problem:(3)

Sparse canonical correlation analysis (SCCA)

In the OCCA, the weight vectors ***α ***and ***β ***are not unique if *p *or *q *exceeds *n*. In addition, it is difficult to interpret the results when there are many non-zero elements in the weight vectors ***α ***and ***β***. In practical applications, especially when *p *and *q *are large, we want to find a linear combination of the weights for **x **and **y **that has large correlation, but that is also sparse for easier interpretation.

To impose the sparsity on ***α ***and ***β***, we propose to consider the following optimization problem with some additional *L*_1 _penalty terms:(4)

where || · ||_1 _is *L*_1 _norm (the sum of all absolute values in the vector), *c*_1 _and *c*_2 _are parameters to control the sparsity and restricted to range 0 <*c*_1 _≤ 1 and 0 <*c*_2 _≤ 1. For simplicity, we use the same value for *c*_1 _and *c*_2 _in this study. The sparse version of CCA is referred to as sparse canonical correlation analysis (SCCA).

The optimization problem in SCCA can be regarded as the problem of penalized matrix decomposition of the matrix *Z *= *X^T^Y*. Recently, a useful algorithm for solving the penalized matrix decomposition (PMD) problem has been proposed and applied to this kind of analysis [34].

The optimisation problem formulated in (4) can be used for finding one canonical component. To extract multiple canonical components, we use a deflation manipulation iteratively as follows:(5)

where *Z *^(*k*) ^is the input of step *k *(*Z *^(1) ^= *X^T^Y *), *d_k _*is the highest singular value, and ***α****_k _*and ***β****_k _*are the weight vectors estimated in the *k*-th step (*k *= 1, 2, ⋯, *m*).

Finally, we obtain *m *pairs of weight vectors ***α***_1_, ⋯, ***α****_m _*and ***β***_1_, ⋯, ***β****_m_*. For easier interpretation, the sign of the weight vectors is adjusted such that the weight element with the highest absolute value is positive in each component. High scoring substructures and side-effects in the weight vectors are extracted as correlated sets.

If the extracted sets of chemical substructures and side-effects are biologically meaningful, potential side-effects for a new drug candidate molecule should be predicted by looking for the extracted chemical substructures in its chemical structure. Suppose that we are given the chemical structure profile **x **of a new drug candidate molecule, and we want to predict its potential side-effect profile **y **based on the extracted sets of chemical substructures and side-effects encoded in  and .

The **x **and **y **are assumed to have their canonical components **u **= *A^T ^***x **and **v **= *B^T ^***y**, respectively, where *A *= [***α***_1_, ⋯, ***α****_m_*], *B *= [***β***_1 _, ⋯, ***β****_m_*]. Since **y **is unknown, we need to estimate **y **such that **v **is close to **u **as much as possible. This estimation can be done by minimizing , which leads to the following solution:(6)

where *B*^-*T *^is the peudo-inverse matrix of *B^T ^*. Note that all data features are normalized in the CCA analysis, each element in the estimate is de-normalized with the standard deviation and the average calculated in the training set. If the *j*-th element in  has a high score, the new molecule **x **is predicted to have the *j*-th side-effect (*j *= 1, 2, ⋯, *q*).

We also consider another prediction score. Based on the weighted sum of canonical components, we propose the following prediction score for a given molecule **x**:(7)

where Λ is the diagonal matrix whose elements are canonical correlation coefficients. Note that *s*(**x**) is the *q*-dimensional vector whose *j*-th element represents a prediction score for the *j*-th side-effect. If the *j*-th element in *s*(**x**) has a high score, the new molecule **x **is predicted to have the *j*-th side-effect (*j *= 1, 2, ⋯, *q*).

In our experience, eq. (7) works similarly as or slightly better than eq. (6), so we use eq. (7) as the prediction score in the result section.

## Authors' contributions

EP - has made substantial contributions to the preparation of the datasets and implementation of the methods, and has also been responsible for drafting the manuscript. VS - has made substantial contributions to the biological interpretations, and has been responsible for drafting some parts of the manuscript. YY - has directed the work, and has been responsible for drafting some parts of the manuscript. All authors read and approved the final manuscript.

## Availability

Project name: Side-effect analysis project; Project home page: http://cbio.ensmp.fr/~yyamanishi/side-effect/; Operating system(s): Platform independent; Programming language: R; Other requirements: "PMA" library in R; Any restrictions to use by non-academics: licence needed.

## Supplementary Material

Additional file 1 **Extracted features by the proposed method**. All the results for drug chemical substructures and drug side-effects extracted by Sparse CCA are summarized in this file. Click here for file

Additional file 2 **Predicted side-effects for unchatecterized drugs in DrugBank**. All the results for uncharacterized drugs in DrugBank are summarized in this file, where the 1st column is Drug ID, the 2nd column is Pubchem compound ID, the 3rd column is the predicted side-effect, and the 4th column is prediction score. Click here for file
